# Low‐Carbohydrate Diet Exacerbates Denervation‐Induced Atrophy of Rat Skeletal Muscle Under the Condition of Identical Protein Intake

**DOI:** 10.1002/jcsm.13738

**Published:** 2025-02-25

**Authors:** Aki Yokogawa, Kohei Kido, Ikuru Miura, Eisuke Oyama, Daisuke Takakura, Keigo Tanaka, Daniel J. Wilkinson, Kenneth Smith, Philip J. Atherton, Kentaro Kawanaka

**Affiliations:** ^1^ Faculty of Sports and Health Sciences Fukuoka University Fukuoka Japan; ^2^ Institute for Physical Activity Fukuoka University Fukuoka Japan; ^3^ Health and Medical Research Institute National Institute of Advanced Industrial Science and Technology (AIST) Takamatsu Japan; ^4^ MRC‐Versus Arthritis Center for Musculoskeletal Ageing Research, Centre of Metabolism, Ageing & Physiology (COMAP), Royal Derby Hospital Center University of Nottingham Derby UK

**Keywords:** AMPK, Atrogin‐1, deuterium oxide, low‐carbohydrate diet, myofibrillar protein synthesis rate

## Abstract

**Background:**

While decreased protein intake is associated with muscle mass loss, it is unclear whether a decrease in carbohydrate intake adversely affects muscle atrophy independently of protein intake. Herein, we examined whether a low‐carbohydrate (low‐CHO) diet exacerbates denervation‐induced muscle atrophy under conditions of identical protein intake.

**Methods:**

On day one of the experiment, male Wistar rats underwent unilateral denervation. The contralateral leg was used as the control. After denervation, rats were divided into two dietary groups: high‐carbohydrate (high‐CHO) and low‐CHO. Each group was fed a high‐CHO (70% carbohydrate) or low‐CHO (20% carbohydrate) diet over 7 days. Total protein and energy intakes in both groups were matched by pair feeding. Rats were provided with deuterium oxide (D_2_O) tracer over the last 3 days of dietary intervention to quantify myofibrillar (muscle) protein synthesis (MPS).

**Results:**

Denervation reduced wet weight of the gastrocnemius muscle compared to the contralateral control (*p* < 0.05). Reductions in gastrocnemius muscle weight were greater in the low‐CHO group (−34%) than the high‐CHO group (−28%) (*p* < 0.05). Although denervation decreased MPS compared to the contralateral control (*p* < 0.05), no dietary effect on MPS was observed. Denervation resulted in increased mRNA and protein expression of Atrogin‐1, a ubiquitin E3 ligase, compared to that in the contralateral control (*p* < 0.05). Increases in Atrogin‐1 gene and protein expression due to denervation were greater in the low‐CHO group than in the high‐CHO group (*p* < 0.05).

**Conclusions:**

We conclude that a low‐CHO diet may exacerbate denervation‐induced atrophy in fast‐twitch‐dominant muscles compared to a high‐CHO diet, even when the same protein intake is maintained. Although blunted MPS contributed to muscle atrophy due to denervation, exacerbation of muscle atrophy by the low‐CHO diet was not accompanied by explanatory changes in MPS. The effect of the low‐CHO diet might be related to promotion of muscle‐specific ubiquitin E3 ligase gene expression.

**Abbreviations**
ACC: acetyl‐CoA carboxylase
AMPK: AMP‐activated protein kinase
ANOVA: analysis of variance
CHO: carbohydrate
FoxO: Forkhead box O
FSR: fractional synthesis rate
LC3: microtubule‐associated protein light chain 3
MPS: muscle protein synthesis
mTORC1: mammalian target of rapamycin complex 1
p70S6K: p70 ribosomal protein S6 kinase
PI3K: phosphoinositide 3‐kinase
SDS‐PAGE: sodium dodecyl sulphate‐polyacrylamide gel electrophoresis
TBS: tris‐buffered saline

## 1 Introduction

Skeletal muscles are the main components of lean body mass and have various metabolic roles beyond locomotion, for example, interorgan flux of substrates. Loss of muscle mass and function has been linked to an increased risk of metabolic diseases such as Type 2 diabetes, poor quality of life, increased morbidity, all‐cause mortality and frailty [1]. Among the mechanisms implicated in muscle atrophy, denervation is one of the most important factors that contributes to muscle wasting during disuse, aging and neurodegenerative diseases [2]. Denervation caused by sciatic nerve transection may be used as an animal model of disuse muscle atrophy and has been used to study the mechanisms of muscle atrophy due to loss of innervation [3, 4, 5].

Theoretically, skeletal muscle atrophy is caused by a decrease in muscle protein synthesis (MPS), an increase in muscle protein breakdown (MPB) or both. On the other hand, short‐term (4 days) human muscle disuse atrophy was reported to be driven by declines in MPS, not increases in MPB [6]. The mammalian target of rapamycin complex 1 (mTORC1) plays an important role in regulating MPS, such as translation initiation and elongation [7]. Contrastingly, ATP‐dependent protein degradation via the ubiquitin–proteasome system is a major proteolytic system that is activated by the expression of the muscle‐specific E3 ubiquitin ligases, for example, Atrogin‐1 (also known as MAFbx) and muscle‐specific ring finger 1 (MuRF‐1) [8].

Dietary nutrients play a fundamental role in maintaining muscle mass in both health and disease. Protein intake, through essential amino acid (EAA) constituents, stimulates MPS and is a primary nonpharmacological factor driving anabolic signals to increase or maintain skeletal muscle mass [9]. Additionally, there is evidence that inadequate protein intake is associated with the age‐related loss of lean mass, particularly muscle mass [10]. Specifically, the EAA, leucine, activates mTORC1 and promotes MPS [9], which may explain how decreased protein intakes can exacerbate muscle atrophy. However, it remains unclear whether carbohydrate intake affects muscle atrophy, even though glucose deficiency downregulates mTORC1 [11, 12].

The low‐CHO diets, such as the ketogenic diet, have a variety of beneficial effects, including weight loss, cancer prevention, glycaemic and lipid control effects and extended longevity and health span [13, 14, 15]. The effect of a low‐CHO diet on muscle mass is controversial. It has been reported to prevent age‐related muscle atrophy [16]. In contrast, a low‐CHO diet has also been reported to decrease muscle mass by reducing MPS [17]. In this previous study, the intervention with a low‐CHO diet, that is, a ketogenic diet, was accompanied by a decrease in protein intake, which suggest the possibility that the decrease in protein intake, but not carbohydrate intake, might have adversely affected muscle mass.

In the present study, we examined the hypothesis that, even if protein intake is maintained at the same level, a low‐CHO diet downregulates the mTORC1 pathway and MPS and exacerbates muscle atrophy due to denervation compared to a high‐CHO diet. Additionally, we examined the effects of a low‐CHO diet on the ubiquitin‐related enzymes, including Atrogin‐1 and MuRF‐1, as crucial factors activating the ubiquitin–proteasome pathway in atrophy scenarios.

## 2 Methods

### 2.1 Animals

Male Wistar rats aged 9 weeks were obtained from CLEA Japan (Tokyo, Japan) and housed in individual cages with controlled temperature (23.5°C ± 0.7°C), humidity (34.0% ± 5.7%) and light (12‐h light–dark cycle). The rats were allowed to acclimatize to the new environment for 1 week following delivery. The Animal Care and Use Committee of Fukuoka University approved this study (Approval Numbers 2104007 and 2112091).

### 2.2 Diet Composition and Protocols

At the end of a 1‐week acclimation period during which the rats were fed a standard laboratory diet, 10‐week‐old rats weighing 300–350 g underwent surgical procedures to induce denervation of the soleus, plantaris and gastrocnemius muscles. Briefly, following sevoflurane anesthetization (4%–5%), the sciatic nerve in the right hind limb was exposed and transected, whereas the left nerve served as a sham‐operated internal control. After administering sterile ampicillin, the incision was sutured and the skin was closed.

Following surgery, rats were randomly divided into two groups: high‐CHO (*n* = 12) and low‐CHO (*n* = 12). The composition of diets, expressed as a percentage of the metabolizable energy (ME), was as follows: high‐CHO diet: 10% fat, 20% protein and 70% carbohydrate (ME 3.85 kcal/g); low‐CHO diet: 60% fat, 20% protein and 20% carbohydrate (ME 5.24 kcal/g). Rodent Diet D12492 (Research Diets, Tokyo, Japan) was used as the high‐CHO diet, and Rodent Diet D12450J (Research Diets, New Brunswick, USA) was used as the low‐CHO diet. The diets were semipurified, and only a single identical source was used for each macronutrient (protein source, sodium casein; fat source, beef tallow; carbohydrate source, starch). Further details of diet composition are provided in Table S1.

Each diet was pair fed on an isoenergetic basis to the corresponding group for 7 days. Pair feeding was performed because in our preliminary ad libitum experiments, rats that were fed a low‐CHO diet consumed approximately 30% more energy than those that were fed a high‐CHO diet (high‐CHO: 607.1 ± 10.1 kcal/week, low‐CHO: 799.5 ± 16.1 kcal/week, *n* = 6 per each group). For the pair‐feeding procedure, daily (ad libitum) food intake of the high‐CHO group was measured, and the daily energy and protein intake was calculated. Each rat in the low‐CHO group was subsequently fed the amount that a single body weight–matched rat in the high‐CHO group had consumed on the previous day. Following the 7‐day pair‐feeding period and after 2 h of fasting the rats were anaesthetized using sevoflurane (4%–5%). The gastrocnemius, plantaris and soleus muscles were dissected from the denervated and contralateral limbs. Additionally, the visceral fat was dissected. The weights of each organ were quantified. The muscles were stored at −80°C until further MPS and biochemical analyses. Blood samples were collected into tubes containing heparin as an anticoagulant, and the plasma was separated via centrifugation at 1750 × *g* for 15 min at 4°C. Plasma aliquots were frozen at −80°C.

### 2.3 Measuring MPS

Three days before muscle collection (4 days after denervation surgery and initiating a low‐ or high‐CHO diet), the animals were administered a D_2_O bolus via oral gavage (7.2 mL/kg, 70 atoms%). The stored muscle samples were prepared to isolate myofibrillar proteins as previously described, with some minor modifications [18]. Briefly, the myofibrillar protein was extracted from approximately 40 mg of muscle tissue. After releasing protein‐bound amino acids using acid hydrolysis, the amino acids were subsequently derivatized as their *n*‐methoxycarbonyl methyl esters. Incorporating deuterium into protein‐bound alanine was determined using gas chromatography–pyrolysis–isotope ratio mass spectrometry (Delta V Advantage, Thermo, Hemel Hempstead, UK) [19]. Plasma D_2_O precursor enrichment was measured as previously described [18]. Myofibrillar MPS was calculated as described in a previous study [20]. Further details of sample preparation and calculations are provided in the Supporting Information.

### 2.4 Western Blot Analysis

Muscle samples for western blotting were prepared and subjected to sodium dodecyl sulphate‐polyacrylamide gel electrophoresis (SDS‐PAGE) and immunoblotting as previously described, with some minor modifications [21, 22]. Further details are provided in Supporting Information. The information on primary antibodies are shown in Table S2.

### 2.5 Real‐Time Polymerase Chain Reaction (PCR) Analysis

After extraction of RNA from gastrocnemius muscle samples, cDNA was synthesized using a PrimeScript RT Reagent Kit (Takara Bio, Shiga, Japan). Synthesized cDNA was used for qPCR using SYBR Green Master Mix (Thermo Fisher Scientific) with the Step One Real‐Time PCR system (Applied Biosystems, MA). The primer sequences are shown in Table S3.

### 2.6 Skeletal Muscle Glycogen and Protein Concentrations

The frozen muscles were weighed and homogenized with 0.3‐M perchloric acid. Glycogen concentrations were determined using the enzymatic method after the acid hydrolysis of glycogen [23]. Frozen muscles were weighed and homogenized in ice‐cold RIPA buffer. The total protein content of the extracts was determined using the BCA assay.

### 2.7 The 20S Proteasome Activity

The frozen muscles were homogenized with 0.5% NP‐40. The samples were centrifuged at 12 000 × *g* for 10 min at 4°C. The supernatants were collected, and 20S proteasome activity was measured using a Proteasome Activity Assay Kit (ac107921; abcam, MA, USA) according to the manufacturer's protocol.

### 2.8 Plasma Sample Analysis

Plasma insulin levels were determined using a rat insulin enzyme‐linked immunosorbent assay kit (Shibayagi, Gunma, Japan) according to the manufacturer's instructions.

### 2.9 Statistical Analyses

Data are presented as the means ± standard deviation of the mean. Two‐way analysis of variance (ANOVA) with repeated measures was used to assess the significant effects of denervation and diet on the outcome variables. A Bonferroni post hoc test was performed for multiple comparisons when an interaction between denervation and diet was observed. Comparisons between two groups were made using Student's unpaired *t* test, which was also used to compare denervation‐induced changes in the sham‐operated contralateral leg between the high‐ and low‐CHO groups. When data from the high‐ and low‐CHO groups in each muscle were combined, a one‐way ANOVA with repeated measures followed by Bonferroni's multiple comparison test was used to determine significant differences between muscle types. The correlations were assessed using Pearson's product–moment correlation coefficients. Statistical significance was set at *p* < 0.05.

## 3 Results

### 3.1 Energy and Nutrient Intake, Body Weight, Visceral Fat and Plasma Insulin

The total energy intake during the 7‐day dietary intervention and denervation period did not differ between the groups (Table 1), because the high‐ and low‐CHO diets were isoenergetically pair fed to the corresponding groups. No difference was observed in the total protein intake between the high‐ and low‐CHO groups (Table 1). Total fat and carbohydrate intakes significantly differed between the groups (*p* < 0.05, Table 1).

**TABLE 1:** The effect of dietary carbohydrate ratio on total energy intake, macronutrients intake, body weight, visceral fat and plasma insulin.

	High‐CHO	Low‐CHO
Total energy intake (kcal/week)	424.1 ± 58.1	423.9 ± 58.1
Total protein intake (kcal/week)	84.8 ± 11.6	84.8 ± 11.6
Total fat intake (kcal/week)	42.4 ± 5.8	254.4 ± 34.9*
Total carbohydrate intake (kcal/week)	296.9 ± 40.7	84.8 ± 11.6*
Body weight (g)	320.7 ± 20.1	313.9 ± 12.2
Epididymal fat weight (mg)	3441.8 ± 443.7	3809.7 ± 567.8
Plasma insulin (ng/mL)	0.78 ± 0.2	1.00 ± 0.80

*Note:* Values are mean ± SD, and all data are from *n* = 12 animals per each of the high‐CHO or low‐CHO group.

Abbreviation: CHO, carbohydrate.

* *p* < 0.05 (unpaired *t* test).

No differences were observed in the body weight or epididymal fat weight between the high‐ and low‐CHO groups (Table 1). Additionally, plasma insulin levels 2 h after initiating fasting did not differ between the groups (Table 1).

### 3.2 Muscle Weight

**FIGURE 1:**
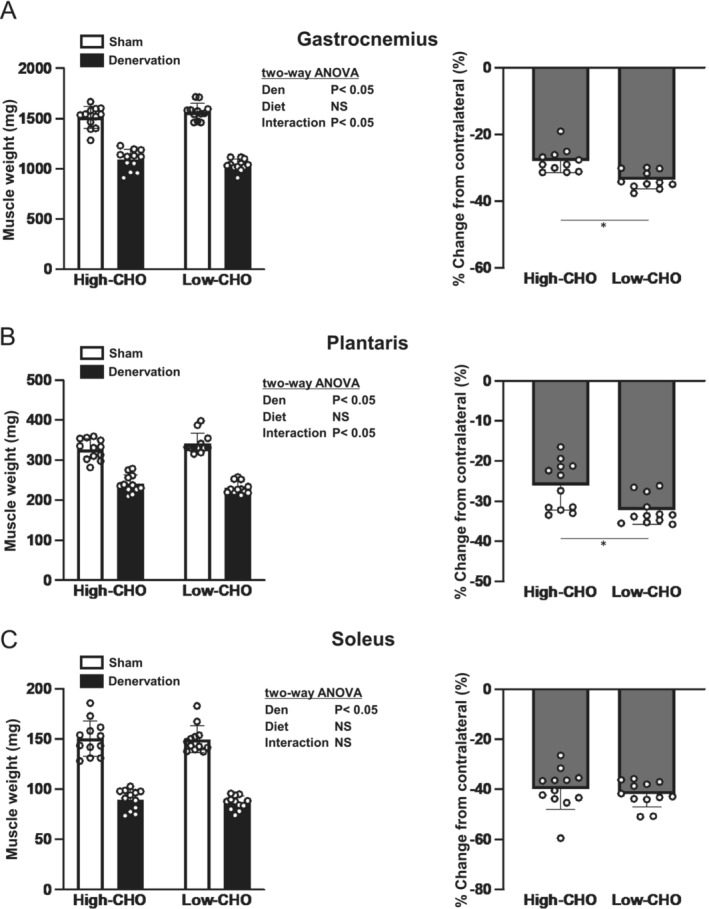
Effects of denervation and dietary carbohydrate ratio on muscle wet weight. Absolute muscle wet weight and denervation‐induced changes from the sham‐operated contralateral leg in gastrocnemius (A), plantaris (B) and soleus muscle (C). Values are expressed as mean ± SD, and all data are from *n* = 12 animals per each of the high‐ or low‐CHO group. Two‐way ANOVA with repeated measures was used to assess the significant effect of denervation and diet. *p < 0.05 (unpaired *t* test). CHO, carbohydrate; Den, denervation; NS, not significant; Sham, sham‐operated contralateral.

Denervation decreased the wet weights of the gastrocnemius, plantaris and soleus muscles (Figure 1A–C). Although no main effect of diet was observed, significant interactions between denervation and diet were observed in the gastrocnemius and plantaris muscles but not in the soleus muscle (Figure 1A–C). The percentage of wet weight loss in the gastrocnemius muscle due to denervation was 28.0% ± 3.3% and 33.7% ± 2.5% in the high‐ and low‐CHO groups, respectively (Figure 1A). The wet weight loss of the plantaris muscle due to denervation was 26.1% ± 5.8% and 32.3% ± 3.4%, respectively (Figure 1B). Weight loss in the gastrocnemius and plantaris muscles due to denervation was greater in the low‐CHO group than that in the high‐CHO group (*p* < 0.05; Figure 1A,B). Contrastingly, wet weight loss of the soleus muscle due to denervation was 39.8% ± 7.9% and 41.9% ± 4.9% in the high‐ and low‐CHO groups, respectively (Figure 1C). No significant differences were observed in the wet weight loss of the soleus muscles between the high‐ and low‐CHO groups (Figure 1C).

### 3.3 MPS Rate

**FIGURE 2:**
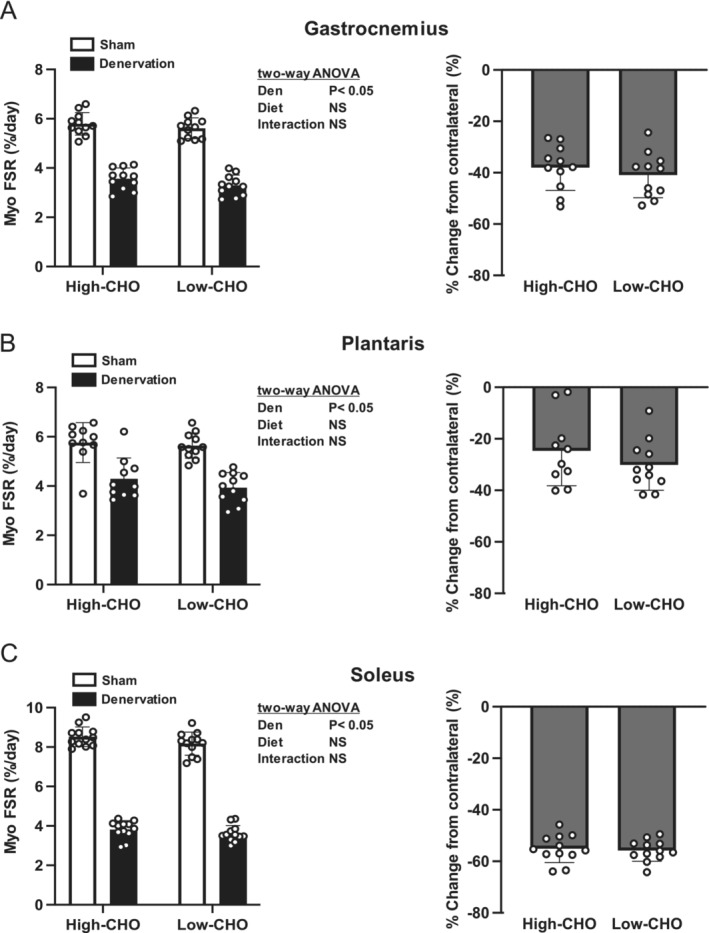
Effects of denervation and dietary carbohydrate ratio on muscle protein synthesis. Absolute myofibrillar fractional synthesis rate and denervation‐induced changes from the sham‐operated contralateral leg in the gastrocnemius (A), plantaris (B) and soleus (C) muscles. Values are expressed as mean ± SD, and data are from *n* = 11 animals for gastrocnemius (A), *n* = 10 or 11 for plantaris (B) and *n* = 12 for soleus (C) per each of the high‐ or low‐CHO group. Two‐way ANOVA with repeated measures was used to assess the significant effect of denervation and diet. CHO, carbohydrate; Den, denervation; MPS, myofibrillar (muscle) fractional protein synthesis rate; NS, not significant; Sham, sham‐operated contralateral.

Denervation decreased the MPS rate of the gastrocnemius, plantaris and soleus muscles (Figure 2A–C). No main effect of diet or its interaction with denervation were observed in any of the muscle types tested (Figure 2A–C). The reduction in the MPS rate in the gastrocnemius muscle due to denervation was 38.1% ± 8.4% and 41.0% ± 8.4% in the high‐ and low‐CHO groups, respectively (Figure 2A). The reduction in the MPS rate in the plantaris muscle was 24.7% ± 12.9% and 30.2% ± 9.3% in the high‐ and low‐CHO groups, respectively (Figure 2B). These values were 55.1% ± 5.1% and 55.8% ± 3.9%, respectively, in the soleus muscle (Figure 2C).

### 3.4 Muscle Weight and Protein Synthesis Rate in Different Types of Muscle

**FIGURE 3:**
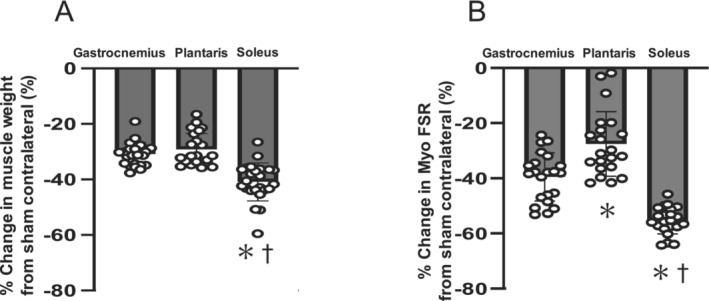
Effects of muscle type on the percentage of muscle wet weight loss and muscle protein synthesis due to denervation. Percentage reductions in muscle wet weight (A) and myofibrillar fraction synthesis rate (B) due to denervation in the gastrocnemius, plantaris and soleus muscles were calculated by combining data from the high‐ and low‐CHO groups. Values are expressed as mean ± SD, and data were obtained from *n* = 24 (A) or 21–24 (B) muscles. One‐way ANOVA with repeated measures followed by Bonferroni multiple comparison test was used to see significant effects of muscle type. *p < 0.05 versus gastrocnemius muscle, ^†^ *p* < 0.05 versus plantaris muscle.

When the data from the high‐CHO and low‐CHO groups for each muscle type were combined, the percentage of wet weight loss due to denervation was greater in the soleus muscle than in the gastrocnemius and plantaris muscles (Figure 3A). The percentage of MPS reduction due to denervation was also greater in the soleus muscle than in the gastrocnemius and plantaris muscles (Figure 3B).

When the data for the high‐CHO and low‐CHO groups were plotted together, no significant positive correlation was observed between the decrease in MPS and muscle weight loss due to denervation in the gastrocnemius, plantaris and soleus muscle (Figure S1A–C). On the other hand, when the data for these muscle types were plotted together, a significant positive correlation was observed between the decrease in MPS and muscle weight loss due to denervation (Figure S1D; *r* = 0.616, *p* < 0.05).

### 3.5 The mTORC1 and Akt Signalling

**FIGURE 4:**
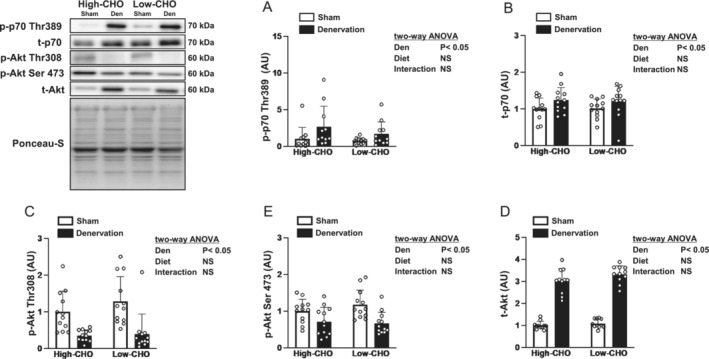
Effects of denervation and dietary carbohydrate ratio on the intramuscular p70S6K and Akt signalling markers in the gastrocnemius muscle. Relative change compared with the sham‐operated contralateral side of the high‐CHO group in phosphorylated p70S6K at Thr389 (A), total p70S6K (B), phosphorylated Akt at Thr308 (C), phosphorylated Akt at Ser473 (D) and total Akt (E) in the gastrocnemius muscle. Values are expressed as mean ± SD, and the data are from *n* = 11 (A,E) or 12 (B–D) muscles per each of the high‐ or low‐CHO groups. Two‐way ANOVA with repeated measures was used to assess the significant effect of denervation and diet. Representative western blots are indicated on the left. CHO, carbohydrate; Den, denervation; NS, not significant; Sham, sham‐operated contralateral.

The main downstream target of mTORC1 is p70 ribosomal protein S6 kinase (p70S6K), which regulates cellular translation. Phosphorylated and total p70S6K were substantially increased in the denervated gastrocnemius muscles compared to the contralateral sham‐operated muscles (Figure 4A,B). No main effect of diet or its interaction with denervation were observed on the phosphorylated and total p70S6K in the gastrocnemius muscle (Figure 4A,B).

The phosphorylated Akt levels at Thr308 and Ser473 were lower, and total Akt level was higher in the denervated gastrocnemius muscle than in the contralateral sham‐operated muscle (Figure 4C–E). No main effect of diet or its interaction with denervation were observed for phosphorylated and total Akt in the gastrocnemius muscle (Figure 4C–E).

### 3.6 Muscle Protein Degradation Signalling Pathway

**FIGURE 5:**
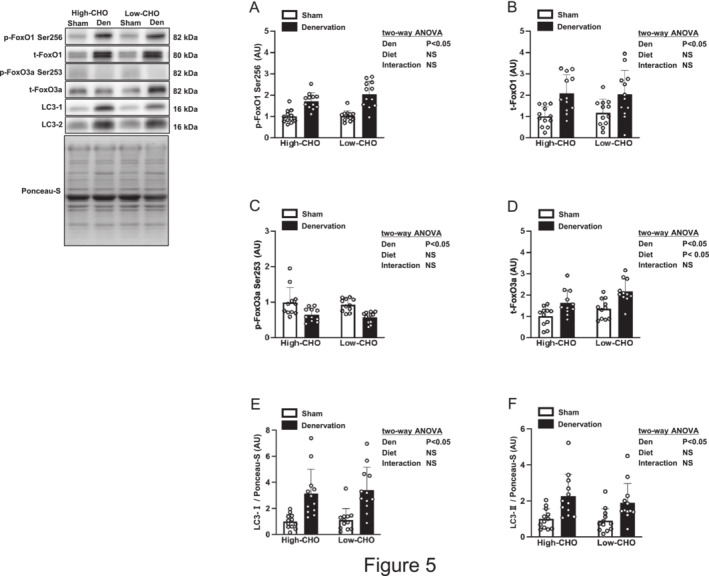
Effects of denervation and dietary carbohydrate ratio on the intramuscular signalling markers for protein breakdown in the gastrocnemius muscle. Relative change compared with the sham‐operated contralateral side of the high‐CHO group in phosphorylated FoxO1 at Ser256 (A), total FoxO1 (B), phosphorylated FoxO3a at Ser253 (C), total FoxO3a (D), LC3‐I (E) and LC3‐II (F) in the gastrocnemius muscle. The data for LC3‐I and LC3‐II were normalized by Ponceau S staining signalling. Values are expressed as mean ± SD, and the data are from *n* = 12 (A,B,E,F) or 11 (C,D) muscles per each of the high‐ or low‐CHO groups. Two‐way ANOVA with repeated measures was used to assess the significant effect of denervation and diet. Representative western blots are indicated on the left. CHO, carbohydrate; Den, denervation; FoxO, Forkhead box O; LC, microtubule‐associated protein light chain; NS, not significant; Sham, sham‐operated contralateral.

Denervation increased FoxO1 phosphorylation at the Akt‐dependent serine site (Ser256) but suppressed FoxO3a phosphorylation at the Akt‐dependent serine site (Ser253) in the gastrocnemius muscle (Figure 5A,C). The total FoxO1 and FoxO3a amounts were upregulated in the gastrocnemius muscle in response to denervation (Figure 5B,D). The low‐CHO diet increased the total FoxO3a level, but not the FoxO1 level, in the gastrocnemius muscle compared with the high‐CHO diet (Figure 5B,D). No interaction between denervation and diet was found on total and phosphorylation levels of FoxOs.

The autophagosomal precursor protein LC3‐I and autophagosomal mature protein LC3‐II were more abundant in the denervated gastrocnemius muscles than in the innervated muscles (Figure 5E,F). No main effect of diet or its interaction with denervation on LC3‐I and LC3‐II were observed in the gastrocnemius muscle (Figure 5E,F).

**FIGURE 6:**
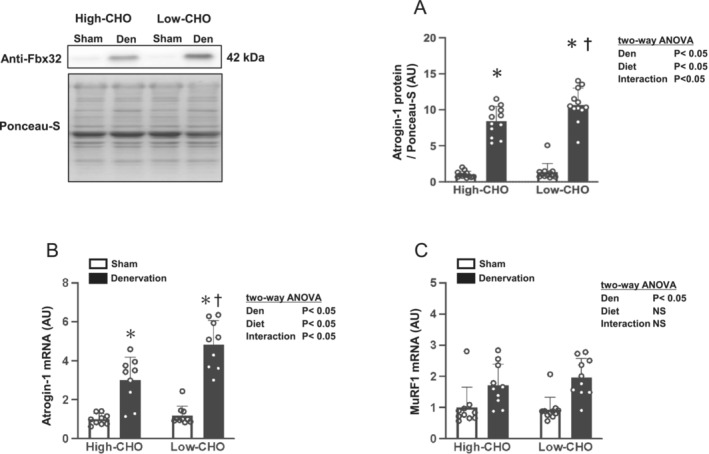
Effects of denervation and dietary carbohydrate ratio on the markers for ubiquitin–proteasome pathway in the gastrocnemius muscle. Relative change compared with the sham‐operated contralateral of the high‐CHO group in Atrogin‐1 protein (A), Atrogin‐1 mRNA (B) and MuRF‐1 mRNA (C) in the gastrocnemius muscle. The data for Atrogin‐1 protein were normalized by Ponceau S staining signalling. Values are expressed as mean ± SD, and the data are from *n* = 12 (A), 9–10 (B) or 10–11 (C) muscles for each high‐ or low‐CHO group. Two‐way ANOVA with repeated measures was used to assess the significant effect of denervation and diet. *p < 0.05 versus Sham (denervation effect using Bonferroni multiple comparison test) and ^†^ *p* < 0.05 versus high‐CHO (diet effect using Bonferroni multiple comparison test). Representative western blots are indicated on the left. CHO, carbohydrate; Den, denervation; NS, not significant; Sham, sham‐operated contralateral.

Denervation increased Atrogin‐1 protein expression level in the gastrocnemius muscle (Figure 6A). Denervation also increased the mRNA expression levels of Atrogin‐1 and MuRF‐1 (Figure 6B,C). Besides, Atrogin‐1 protein and mRNA levels in the denervated gastrocnemius muscles were significantly higher in the low‐CHO than in the high‐CHO diet groups (*p* < 0.05, Figure 6A,B).

Figure S2 illustrates the mRNA expression levels of other E3 ubiquitin ligases, that is, MUSA1 and SMART, and deubiquitinating enzymes, that is, USP19. Denervation increased the mRNA expression level of MUSA1 in the gastrocnemius muscle but suppressed the expression levels of SMART and USP19 (Figure S2A). No main effect of diet or its interaction with denervation on the mRNA levels of these ubiquitin‐related enzymes were observed in the gastrocnemius muscle (Figure S2A).

Figure S2 also illustrates the total ubiquitinated protein level and 20S proteasome activity. The total ubiquitinated protein level was higher in the denervated gastrocnemius muscle than in the contralateral sham‐operated muscle, and 20S proteasome activity was lower (Figure S2A). No main effect of diet or its interaction with denervation were observed for total ubiquitinated protein level and 20S proteasome activity in the gastrocnemius muscle (Figure S2A).

### 3.7 AMP‐Activated Protein Kinase (AMPK) Signalling Pathway

**FIGURE 7:**
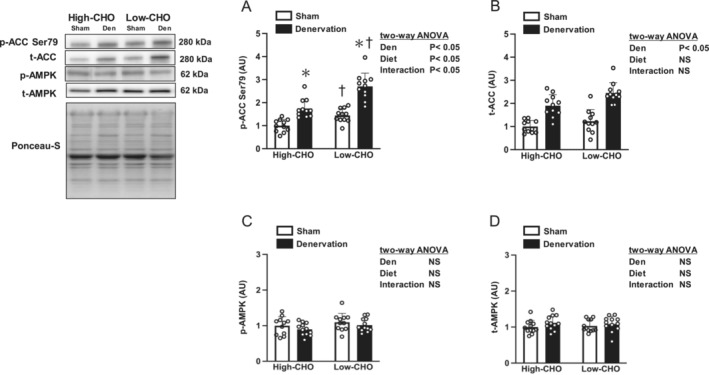
Effects of denervation and dietary carbohydrate ratio on the intramuscular AMPK signalling markers in the gastrocnemius muscle. Relative change compared with the sham‐operated contralateral of the high‐CHO group in phosphorylated ACC at Ser79 (A), total ACC (B), phosphorylated AMPK at Thr308 (C) and total AMPK (D) in the gastrocnemius muscle. Values are expressed as mean ± SD, and the data are from *n* = 11–12 (A–D) muscles per each of the high‐ or low‐CHO groups. Two‐way ANOVA with repeated measures was used to assess the significant effect of denervation and diet. *p < 0.05 versus Sham (denervation effect using Bonferroni multiple comparison test) and ^†^ *p* < 0.05 versus high‐CHO (diet effect using Bonferroni multiple comparison test). Representative western blots are indicated on the left. ACC, acetyl‐CoA carboxylase; CHO, carbohydrate; Den, denervation; NS, not significant; Sham, sham‐operated contralateral.

The phosphorylation level of ACC, a downstream substrate of AMPK, is a marker of AMPK activation, indicating that AMPK activation was higher in the denervated gastrocnemius muscle than in the contralateral sham‐operated muscle (Figure 7A). Additionally, ACC phosphorylation in the denervated gastrocnemius muscles was significantly higher in the low‐CHO than in the high‐CHO diet group (*p* < 0.05; Figure 7A). Although denervation increased the total ACC in the gastrocnemius muscle, no main effect of diet or interaction between denervation and diet were observed for the total ACC in the gastrocnemius muscle (Figure 7B).

### 3.8 Muscle Glycogen and Protein Concentrations

As shown in Table S4, in the gastrocnemius and plantaris muscle, denervation caused a decrease in the total protein concentration. However, no main effect of diet or its interaction with denervation on the total protein concentration were observed. No main effects of denervation and diet or their interaction were observed in the soleus muscle.

As shown in Table S6, in the gastrocnemius, plantaris and soleus muscles, denervation caused a decrease in muscle glycogen. In the gastrocnemius muscle, no main effect of diet was observed for glycogen. However, the low‐CHO diet decreased the levels of plantaris and soleus muscle glycogen compared to the high‐CHO diet. No interactions between denervation and diet were observed in the gastrocnemius, plantaris and soleus muscles.

## 4 Discussion

First, this study exposes that the low‐CHO diet exacerbated denervation‐induced atrophy of fast‐twitch dominant muscles (gastrocnemius and plantaris) compared to the high‐CHO diet, even at the same protein and energy intake. Second, that a low‐CHO diet did not attenuate denervation‐induced decreases in MPS but promoted the denervation‐induced increases in gene and protein expression of the muscle‐specific ubiquitin E3 ligase, Atrogin‐1. In other words, reducing carbohydrate intake may adversely affect muscle atrophy due to denervation independently of protein intake. Increased expression of Atrogin‐1 might be involved in the promotion of denervation muscle atrophy by a low‐CHO diet.

Herein, denervation caused atrophy of the gastrocnemius, plantaris and soleus muscles; however, greater atrophy was observed in the soleus muscles. This is consistent with a previous study that demonstrated the soleus muscle to be more susceptible to denervation [3]. The soleus is a weight‐bearing muscle that has a substantial proportion of Type I fibres and few Type II fibres. Contrastingly, the gastrocnemius and plantaris muscles are Type II fibre dominant [24]. Type I fibres are heavily recruited (~7 h/day) during daily physical activities, whereas Type II fibres are rarely recruited [25], which may explain why the soleus muscle is more susceptible to atrophy due to denervation. Expectedly, denervation decreased myofibrillar MPS in all muscles in the present study. With ~70% of muscle proteins estimated to be myofibrillar, it follows that losses in myofibrillar proteins were largely responsible for the changes in muscle protein mass [26]. Therefore, a decrease in myofibrillar MPS may have contributed to the denervation‐induced muscle atrophy observed herein. This supports the notion that decreases in MPS are the dominant mechanism of disuse, as has also been reported in humans [6, 27]. In further support of this, the atrophy due to denervation being greatest in the soleus muscle can be explained by the reduction in MPS also being the greatest, because the percentage reduction in muscle weight and MPS due to denervation was greater in the soleus muscle than in the gastrocnemius or plantaris muscles.

Activation of mTORC1 signalling is typically implicated in enhancing MPS and maintaining muscle mass [7]. However, the present study demonstrated that, despite decreases in MPS and muscle mass, denervation increased S6K phosphorylation, consistent with results of previous studies [4, 5]. Chronic mTORC1 activation and subsequent S6K activation leads to IRS1 phosphorylation and PI3K‐Akt pathway inhibition [28]. Inhibition of Akt may promote the entry of FoxOs into the nucleus and increase the transcription of muscle‐specific ubiquitin ligases, such as Atrogin‐1, MuRF‐1 and MUSA1, as well as autophagy‐related proteins, such as LC3 [5, 29]. They may lead to the enhanced protein degradation and muscle atrophy. Indeed, in the current study, we observed decreased Akt phosphorylation, decreased FoxO3a phosphorylation at the Akt‐dependent serine site (Ser253) and increased expression of the atrophy‐related genes noted above (Atrogin‐1, MuRF‐1, MUSA1 and LC3) in denervated muscle.

In the present study, a low‐CHO diet exacerbated denervation‐induced atrophy in fast‐twitch‐dominant muscles, that is, gastrocnemius and plantaris muscles, even under conditions of identical protein intake. Thus, decreased carbohydrate intake may adversely affect muscle atrophy independent of protein intake. In cell culture systems, such as HEK‐293T cells, glucose deprivation inhibits mTORC1 in an AMPK‐dependent or AMPK‐independent pathway [11, 12]. In isolated rat skeletal muscles, glucose itself was also reported to stimulate MPS [30, 31]. Therefore, we expected that by suppressing MPS, a low‐CHO diet exacerbates denervation atrophy. However, dietary carbohydrate ratio did not mitigate the decrease in MPS in denervated muscles.

Contrastingly, our low‐CHO diet promoted the denervation‐induced increase in ACC phosphorylation, a marker for AMPK activation. AMPK has been reported to phosphorylate the transcription factor, FoxO3a, at the Akt‐independent serine and threonine sites (Thr179, Ser399, Ser413, Ser555, Ser588 and Ser626) to stimulate its transcriptional activity [32]. This promotes FoxO3a‐dependent transcription of atrophy‐related genes, that is, the E3 ubiquitin ligases, Atrogin‐1 and MuRF‐1, which are important regulators of the ubiquitin–proteasome pathway [33, 34]. Indeed, our low‐CHO diet promoted the denervation‐induced gene and protein expression of Atrogin‐1, a downstream molecule of FoxO3a. Herein, our low‐CHO diet tended to further decrease muscle glycogen content. It is well known that AMPK binds directly to glycogen via the carbohydrate‐binding domain of the β‐subunit [35] and that there is a reciprocal relationship between reduced muscle glycogen content and higher AMPK activity in skeletal muscle [36]. Thus, a low‐CHO diet may have increased AMPK activity and promoted atrophy‐related gene expression by lowering muscle glycogen content. This might contribute to the enhanced muscle protein degradation and exacerbated muscle atrophy due to denervation.

However, there are some limitations to the above speculation regarding the mechanism by which low‐CHO diet exacerbates muscle atrophy. First, it should be noted that AMPK phosphorylation, another marker of AMPK activation, did not change in response to denervation and diet. It has been reported that AMPK phosphorylation does not always reflect AMPK activation [37]. In addition, ACC phosphorylation is sometimes more sensitive than AMPK phosphorylation in assessing AMPK activation [37]. We therefore measured ACC phosphorylation as an indicator of the AMPK activity, but it has also been reported that ACC is phosphorylated by another kinase in addition to AMPK [38]. Thus, ACC and AMPK phosphorylation does not appear to be a perfect marker for AMPK activation. Further studies are needed to examine the possibility that low‐CHO diet exacerbates muscle atrophy through AMPK activation. Second, our low‐CHO diet promoted denervation‐induced gene and protein expression of the E3 ubiquitin ligase, that is, Atrogin‐1, but did not affect the expression of other ubiquitin ligases, that is, MuRF‐1 and MUSA1, and deubiquitinating enzymes, that is, USP19. In addition, it did not affect the 20S proteasome activity that degrades polyubiquitinated proteins into peptides. The currently available information regarding the ubiquitin–proteasome pathway provides no conclusion regarding possible mechanisms for the exacerbated effect of low‐CHO diet on muscle atrophy, making it an interesting topic for future research.

In this study, the animals were fed diets with varying carbohydrate and fat ratios while maintaining a constant protein ratio. We observed that the lower the ratio of carbohydrates in the diet, the greater the muscle atrophy, and reciprocally, the higher the ratio of fat in the diet, the greater the muscle atrophy (due to denervation). Roseno et al. reported that a 3‐week high‐fat diet augmented denervation muscle atrophy by increasing protein degradation via the ubiquitin–proteasome pathway [39]. Because a high‐fat diet accompanies excess caloric intake, this previous study suggested that excessive energy intake and the associated obesity may exacerbate muscle atrophy. Contrastingly, in the present study, high‐CHO (low‐fat) and low‐CHO (high‐fat) diet groups were compared by pair feeding to ensure the same energy intake. In addition, no differences in visceral fat were observed between the two groups. Thus, factor(s) derived from the differences in the dietary ratio of carbohydrates to fat, other than energy intake and obesity, influence our results.

The low‐CHO diet exacerbated the denervation‐induced atrophy of fast‐twitch muscles, namely, gastrocnemius and plantaris muscles, but not that of slow‐twitch muscles, namely, soleus muscles. Consistent with the present study findings, it has been reported that fast‐twitch muscle atrophy is more sensitive to nutrient deprivation than slow‐twitch muscle atrophy [40]. Our study and the previous study suggest that there is a regulatory system for muscle mass related to nutrients such as carbohydrates and that this system may play a more important role in fast‐twitch muscles than in slow‐twitch muscles. Slow‐twitch muscles are weight‐bearing muscles highly recruited during daily physical activities, whereas fast‐twitch muscles are rarely recruited. Thus, fast‐twitch muscles appear to function in daily life as the primary protein store rather than as a locomotive device. For this reason, when faced with carbohydrate deprivation, the breakdown of protein stores may be accelerated more in fast‐twitch muscles than in slow‐twitch muscles. This is a mechanism that supplies amino acids as materials for gluconeogenesis. Thus, fast‐twitch muscles might be more sensitive to atrophy during carbohydrates deprivation.

In conclusion, a 7‐day unilateral denervation caused muscle atrophy in rat gastrocnemius, plantaris and soleus muscles. Blunted MPS contributed to denervation‐induced muscle atrophy. Furthermore, the low‐CHO diet exacerbated denervation‐induced atrophy of fast muscles (gastrocnemius and plantaris) under identical protein and energy intake conditions. Thus, reducing carbohydrate intake may adversely affect muscle atrophy due to denervation independently of protein intake. This exacerbation of muscle atrophy by the low‐CHO diet may not be due to the accelerated decline in MPS. Moreover, it might be involved with the enhanced ubiquitin E3 ligase (Atrogin‐1) gene and protein expression, although there is not sufficient evidence to date. These results highlight the importance of the dietary carbohydrate ratio in maintaining skeletal muscle mass and provide a unique paradigm for establishing dietary strategies that optimally preserve muscle mass for health and survival.

## Ethics Statement

This study was approved by the Animal Care and Use Committee of Fukuoka University (Approval Numbers 2104007 and 2112091).

## Conflicts of Interest

The authors declare no conflicts of interest.

## Supporting information


**Table S1.** Experimental food composition.
**Table S2.** Antibody list.
**Table S3.** Primer sequences.
**Table S4.** The effects of denervation and dietary carbohydrate ratio on muscle protein concentration.
**Table S5.** The effects of denervation and dietary carbohydrate ratio on total muscle protein content.
**Table S6.** The effects of denervation and dietary carbohydrate ratio on muscle glycogen concentration.

**Figure S1.** Correlation between decreased muscle protein synthesis and muscle weight loss due to denervation.
**Figure S2.** Effects of denervation and dietary carbohydrate ratio on the total ubiquitinated protein, ubiquitin‐related enzyme mRNA and 20S proteasome activity in the gastrocnemius and soleus muscle.
**Figure S3.** Effects of denervation and dietary carbohydrate ratio on the intramuscular p70S6K and Akt signalling markers in the soleus muscle.
**Figure S4.** Effects of denervation and dietary carbohydrate ratio on the intramuscular signalling markers for protein breakdown in the soleus muscle.
**Figure S5.** Effects of denervation and dietary carbohydrate ratio on the markers for ubiquitin–proteasome pathway in the soleus muscle.
**Figure S6.** Effects of denervation and dietary carbohydrate ratio on intramuscular AMPK signalling markers in the soleus muscle.
