# Supplementary material for: Low‐Carbohydrate Diet Exacerbates Denervation‐Induced Atrophy of Rat Skeletal Muscle Under the Condition of Identical Protein Intake
Source: J Cachexia Sarcopenia Muscle. 2025 Feb 25;16(2):e13738. doi: 10.1002/jcsm.13738 (PMC11859665; doi:10.1002/jcsm.13738)
Supplement: Supplementary file 1 — Table S1. Experimental food composition. Table S2. Antibody list. Table S3. Primer sequences. Table S4. The effects of denervation and dietary carbohydrate ratio on muscle protein concentration. Table S5. The effects of denervation and dietary carbohydrate ratio on total muscle protein content. Table S6. The effects of denervation and dietary carbohydrate ratio on muscle glycogen concentration. [file JCSM-16-e13738-s001.pdf]

Supplementary Information

Article titled "Low-carbohydrate diet exacerbates denervation-induced atrophy of rat skeletal muscle under the condition of identical protein intake" for publication in *J Cachexia Sarcopenia Muscle*.

The paper was coauthored by Aki Yokogawa, Kohei Kido, Ikuru Miura, Eisuke Oyama, Daisuke Takakura, Keigo Tanaka, Daniel J. Wilkinson, Kenneth Smith, Philip J. Atherton, Kentaro Kawanaka.

Supplementary Methods ········ Page_2
Supplementary References ········ Page_8
Supplementary Table 1 ········ Page_9
Supplementary Table 2 ········ Page_10
Supplementary Table 3 ········ Page_11
Supplementary Table 4 ········ Page_12
Supplementary Table 5 ········ Page_13
Supplementary Table 6 ········ Page_14
Supplementary Figure legends ········ Page_15
Supplementary Figure 1 ········ Page_18
Supplementary Figure 2 ········ Page_19
Supplementary Figure 3 ········ Page_20
Supplementary Figure 4 ········ Page_21
Supplementary Figure 5 ········ Page_22
Supplementary Figure 6 ········ Page_23

Abbreviations: EDTA, ethylenediaminetetraacetic acid; EGTA, ethylene glycol tetraacetic acid; NaF, sodium fluoride; MOPS, 3-(*N*-morpholino)propanesulfonic acid; KCl, potassium chloride; NH_4OH, ammonium hydroxide; D_2O, deuterium oxide; APE, atom percent excess; SDS-PAGE, sodium dodecyl sulfate-polyacrylamide gel electrophoresis; RIPA, radioimmunoprecipitation assay; PMSF, phenylmethylsulfonyl fluoride; BCA, bicinchoninic acid; TBST, Tris-buffered saline containing 0.1% Tween-20; PCR, polymerase chain reaction

Supplementary Methods

Measuring muscle protein synthesis (MPS)

Three days before muscle collection (four days after denervation surgery and initiating a low- or high-CHO diet), the animals were administered a D_2O bolus via oral gavage (7.2 ml/kg, 70 atoms%). The stored muscle samples were prepared to isolate myofibrillar proteins as previously described [1]. Briefly, approximately 40 mg of muscle was homogenized in ice-cold homogenization buffer (50 mM Tris-HCl, pH 7.5, 1 mM EDTA, 1 mM EGTA, 10 mM β-glycerophosphate, and 50 mM NaF), before continuous vortexing for 10 min, and centrifugation at 11,000 × *g* for 15 min at 4℃. The resultant pellet was resuspended in extraction buffer (20 mM MOPS, 110 mM KCl, and 1mM EGTA) and subsequently homogenized using Dounce and centrifuged at 1000 × *g* for 5 min at 4℃. The resultant myofibrillar pellets were solubilized in 0.3 M NaOH, separated from the insoluble collagen via centrifugation, and the myofibrillar protein was precipitated using 1 M perchloric acid. Protein-bound amino acids were released using acid hydrolysis by incubating at 110℃ in 0.1 M HCl in Dowex H+ resin slurry overnight before being eluted from the resin with 2 M NH_4OH and evaporated to dryness; the amino acids were subsequently derivatized as their n-methoxycarbonyl methyl esters. Dried samples were suspended in 60 µl

distilled water and 32 μl methanol, and following vortex, 10 μl of pyridine and 8 μl of methylchloroformate were added. The samples were vortexed for 30 s and allowed to react at room temperature for 5 min. The newly formed n-methoxycarbonyl methyl esters of the amino acids were subsequently extracted into 100 μl of chloroform. A molecular sieve was added for approximately 30 s to remove water before being transferred to vials. Incorporating deuterium into protein-bound alanine was determined using gas chromatography-pyrolysis-isotope ratio mass spectrometry (Delta V Advantage, Thermo, Hemel Hempstead, UK) [2]. Plasma D_2O precursor enrichment was measured as previously described [1]. Briefly, pure fractions of body water were extracted by heating 100 μl of plasma in an inverted 2-ml autosampler vial for 4 h at 100°C. The vials were subsequently placed upright on ice to condense the extracted body water and transferred to a clean autosampler vial ready for injection.

Myofibrillar MPS was calculated from the deuterium enrichment (APE) in alanine in myofibrillar proteins using the enrichment of body water (APE, corrected for the mean number of deuterium moieties incorporated per alanine, 3.7, and the dilution from the total number of hydrogens in the derivative, i.e. 11 as surrogate precursor labeling. The fractional synthetic rate (FSR) was

calculated as follows:

$$FSR (\%/h) = (APE_{Ala})/(APE_{p}) \times 1/t \times 100$$

Where APE_{Ala} is the deuterium enrichment of myofibrillar protein-bound alanine, APE_{p} is the precursor enrichment of body water, and t is the time between the D_2O bolus administration and muscle dissection. To calculate APE_{Ala}, both pre (t=0 h, baseline) and post (t=72 h after D_2O administration, at necropsy) deuterium enrichment of myofibrillar protein-bound alanine is required. However, it is impossible to obtain both pre and post muscle sample from the same animal. We substituted pre enrichment with average enrichment in 4 samples from animals that were not administered D_2O, as described in a previous study [3].

Western blot analysis

Muscle samples for western blotting were prepared and subjected to sodium dodecyl sulfate-polyacrylamide gel electrophoresis (SDS-PAGE) and immunoblotting as previously described, with some minor modifications [4, 5]. Briefly, frozen muscles were homogenized in ice-cold radioimmunoprecipitation

assay (RIPA) buffer (FujiFilm Wako Pure Chemical Corp., Tokyo) containing Protease Inhibitor Cocktail (ProteoGuard™ Protease Inhibitor Cocktail containing 0.8 μM aprotinin, 50 μM bestatin, 20 μM leupeptin, 10 μM pepstatin A, and 1 mM PMSF; Takara, Shiga, Japan) and phosphatase inhibitor cocktail (Nacalai Tesque, Kyoto, Japan). The homogenates were subsequently rotated end-over-end for 60 min at 4°C and centrifuged at 10,000 × *g* for 15 min at 4°C.

Aliquots of the supernatants were used to determine protein concentration using bicinchoninic acid (BCA) assay (Pierce™ BCA Protein Assay Kit, Thermo Fisher Scientific). The supernatants were solubilized in Laemmli sample buffer containing 100 mM dithiothreitol (Bio-Rad) and boiled. Samples (10 μg of protein) were separated via SDS-PAGE using a 7.5, 10, or 12% polyacrylamide gel. The resolved proteins were subsequently transferred to a polyvinylidene difluoride membrane and blocked with 5% fat-free skim milk in Tris-buffered saline containing 0.1% Tween-20 (TBST) at pH 7.5. After blocking for 60 min at room temperature, the membranes were washed in TBST and incubated overnight at 4℃ with the appropriate primary antibody. The information on primary antibodies are shown in Supplementary Table 2. After the membranes were washed, they were further incubated with horseradish

peroxidase-conjugated anti-rabbit IgG (H+L) (Cell Signaling Technology, MA, USA) for 60 min at room temperature). Bound antibodies were detected using ECL Prime Western Blotting Detection Reagent and analyzed using an Amersham Imager 600 (GE Healthcare Life Sciences, Tokyo, Japan). Equal protein concentrations were loaded into each lane and confirmed via Ponceau S staining of the membrane.

Real-time polymerase chain reaction (PCR) analysis

The gastrocnemius muscle samples were homogenized in ISOGEN (NIPPON GENE, Tokyo, Japan), chloroform was added, and the homogenate was centrifuged at 12,000 × *g* for 20 min. The supernatants were mixed with an equal volume of 2-propanol and centrifuged at 12,000 × *g* for 15 min to precipitate total RNA. Ethanol (75%) was added to the precipitate, which was subsequently centrifugated at 7,500 × *g* for 5 min. The supernatant was removed, and the ethanol was completely dried and eluted using ultrapure water. The RNA concentrations and purity were measured using a NanoDrop™ Lite spectrophotometer (Thermo Fisher Scientific, MA, USA). cDNA was synthesized using a PrimeScript RT Reagent Kit (Takara Bio, Shiga, Japan).

Synthesized cDNA was used for qPCR using SYBR Green Master Mix (Thermo Fisher Scientific) with the Step One Real-Time PCR system (Applied Biosystems, MA). The primer sequences are shown in Supplementary Table 3. Results were normalized to the expression of cyclophilin B.

Supplementary References

1. Wilkinson DJ, Franchi MV, Brook MS, Narici MV, Williams JP, Mitchell WK, Szewczyk NJ, Greenhaff PL, Atherton PJ, Smith K. A validation of the application of D(2)O stable isotope tracer techniques for monitoring day-to-day changes in muscle protein subfraction synthesis in humans. Am J Physiol Endocrinol Metab. 2014;306:E571-9.
2. Brook MS, Wilkinson DJ, Mitchell WK, Lund JN, Phillips BE, Szewczyk NJ, Greenhaff PL, Smith K, Atherton PJ. Synchronous deficits in cumulative muscle protein synthesis and ribosomal biogenesis underlie age-related anabolic resistance to exercise in humans. J Physiol. 2016;594:7399-7417.
3. Bass JJ, Kazi AA, Deane CS, Nakhuda A, Ashcroft SP, Brook MS, Wilkinson DJ, Phillips BE, Philp A, Tarum J, Kadi F, Andersen D, Garcia AM, Smith K, Gallagher IJ, Szewczyk NJ, Cleasby ME, Atherton PJ. The mechanisms of skeletal muscle atrophy in response to transient knockdown of the vitamin D receptor in vivo. J Physiol. 2021;599:963-979.
4. Kido K, Egawa T, Fujiyoshi H, Suzuki H, Kawanaka K, Hayashi T. AMPK is indispensable for overload-induced muscle glucose uptake and glycogenesis but dispensable for inducing hypertrophy in mice. FASEB J. 2021;35:e21459.
5. Toyoda T, Hayashi T, Miyamoto L, Yonemitsu S, Nakano M, Tanaka S, Ebihara K, Masuzaki H, Hosoda K, Inoue G, Otaka A, Sato K, Fushiki T, Nakao K. Possible involvement of the alpha1 isoform of 5'AMP-activated protein kinase in oxidative stress-stimulated glucose transport in skeletal muscle. Am J Physiol Endocrinol Metab. 2004;287:E166-73.

Supplementary Table 1: Experimental food composition

	Low-Carbohydrate		High-Carbohydrate	
	gm%	kcal%	gm%	kcal%
Protein	26.0	20	19.2	20
Carbohydrate	26.0	20	67.3	70
Fat	35.0	60	4.3	10
Total		100		100
kcal/gm	5.24		3.85	
	gm	kcal	gm	kcal
Casein, 30 Mesh	200	800	200	800
L-Cystine	3	12	3	12
Corn Starch	0	0	506.2	2024.8
Maltodextrin 10	125	500	125	500
Sucrose	68.8	275	68.8	275.2
Cellulose, BW200	50	0	50	0
Soybean Oil	25	225	25	225
Lard	245	2205	20	180
Mineral Mix S 10026	10	0	10	0
DiCalcium Phosphate	13	0	13	0
Calcium Carbonate	5.5	0	5.5	0
Potassium Citrate, 1 H2O	16.5	0	16.5	0
Vitamin Mix V10001	10	40	10	40
Choline Bitartrate	2	0	2	0
FD&C Yellow Dye #5			0.04	0
FD&C Blue Dye #1	0.05	0	0.01	0
Total	773.85	4057	1055.05	4057

Supplementary Table 2: Antibody list

Antibody	Host	Clonality	Company	Catalog#
Akt	Rabbit	Polyclonal	Cell Signaling Technology	9272
Phospho-Akt Ser473	Rabbit	Polyclonal	Cell Signaling Technology	9271
Phospho-Akt Thr308	Rabbit	Polyclonal	Cell Signaling Technology	9275
p70 S6 Kinase	Rabbit	Monoclonal	Cell Signaling Technology	2708
Phospho-p70 S6 Kinase Thr389	Rabbit	Monoclonal	Cell Signaling Technology	9234
FoxO1	Rabbit	Monoclonal	Cell Signaling Technology	2880
Phospho-FoxO1 Ser256	Rabbit	Polyclonal	Cell Signaling Technology	9461
FoxO3a	Rabbit	Monoclonal	Cell Signaling Technology	2497
Phospho-FoxO3a Ser253	Rabbit	Polyclonal	Cell Signaling Technology	9466
LC3A/B	Rabbit	Polyclonal	Cell Signaling Technology	4108
Acetyl-CoA Carboxylase	Rabbit	Polyclonal	Cell Signaling Technology	3662
Phospho-Acetyl-CoA Carboxylase Ser79	Rabbit	Polyclonal	Cell Signaling Technology	3661
AMPKα	Rabbit	Polyclonal	Cell Signaling Technology	2532
Phospho-AMPKα Thr172	Rabbit	Polyclonal	Cell Signaling Technology	2531
Ubiquitin	Rabbit	Polyclonal	Cell Signaling Technology	58395
Fbx32	Rabbit	Monoclonal	Abcam	ab168372

Supplementary Table 3: Primer sequences

Gene name	Forward	Reverse
Atrogin-1	TGAAGACCGGCTACTGTGGAAGAGAC	TTGGGGTGAAAGTGAGACGGAGCAG
MuRF-1	TCGACATCTACAAGCAGGA	CTGTCCTTGGAAGATGCTTT
MUSA1	TGGCACAAGTCAGAATGCTC	CAGCTTCCACACAGTCTCCA
Fbxo21 / SMART	GCAGCAGCCAGATGACTACG	TTTGCACACAAGCTCCACGA
USP19	GTGTCCACGTGAAGCCTGAG	CTCAAAGCACAGGAGCACGT
Cyclophilin B	AGACGAACCTGTAGGACGAG	AGATGCTCTTTCCTCCTGTG

Supplementary Table 4: The effects of denervation and dietary carbohydrate ratio on muscle protein concentration

		High-CHO	Low-CHO	Main effect of Den	Main effect of Diet	Interaction
Gastrocnemius (mg/g tissue)	Sham	131.2 ± 5.7	127.2 ± 7.7	p<0.05	NS	NS
	Denervated	121.2 ± 8.6	116.5 ± 8.3			
Plantaris (mg/g tissue)	Sham	126.7 ± 12.6	128.1 ± 14.3	p<0.05	NS	NS
	Denervated	117.6 ± 15.0	109.8 ± 8.8			
Soleus (mg/g tissue)	Sham	82.2 ± 19.1	85.0 ± 19.5	NS	NS	NS
	Denervated	86.9 ± 15.6	87.5 ± 19.6			

Values are mean ± SD, and all data are from n = 12 animals per each of the high-CHO or low-CHO group. Two-way ANOVA with repeated measures was used to assess the significant effect of denervation and diet. Sham, sham-operated contralateral; Den, denervation; CHO, carbohydrate.

Supplementary Table 5: The effects of denervation and dietary carbohydrate ratio on total muscle protein content

		High-CHO	Low-CHO	Main effect of Den	Main effect of Diet	Interaction
Gastrocnemius (mg/muscle)	Sham	198.1 ± 12.4	199.5 ± 18.2	p<0.05	NS	NS
	Denervated	132.1 ± 14.7	120.9 ± 10.9			
Plantaris (mg/muscle)	Sham	41.3 ± 4.4	44.0 ± 7.6	p<0.05	NS	p<0.05
	Denervated	28.3 ± 3.9	25.3 ± 2.0			
Soleus (mg/muscle)	Sham	12.4 ± 3.1	12.7 ± 3.2	p<0.05	NS	NS
	Denervated	7.9 ± 2	7.6 ± 1.8			

Values are mean ± SD, and all data are from n = 12 animals per each of the high-CHO or low-CHO group. Two-way ANOVA with repeated measures was used to assess the significant effect of denervation and diet. Sham, sham-operated contralateral; Den, denervation; CHO, carbohydrate.

Supplementary Table 6: The effects of denervation and dietary carbohydrate ratio on muscle glycogen concentration

		High-CHO	Low-CHO	Main effect of Den	Main effect of Diet	Interaction
Gastrocnemius (μ mol/g tissue)	Sham	33.1 ± 7.1	31.4 ± 5.2	p<0.05	NS	NS
	Denervated	19.0 ± 1.8	16.6 ± 4.1			
Plantaris (μ mol/g tissue)	Sham	31.7 ± 4.3	29.2 ± 3.4	p<0.05	p<0.05	NS
	Denervated	18.0 ± 4.4	13.6 ± 2.8			
Soleus(μ mol/g tissue)	Sham	29.1 ± 7.5	23.1 ± 2.4	p<0.05	p<0.05	NS
	Denervated	16.3 ± 2.6	15.2 ± 2.6			

Values are mean ± SD, and all data are from n = 12 animals per each of the high-CHO or low-CHO group. Two-way ANOVA with repeated measures was used to assess the significant effect of denervation and diet. Sham, sham-operated contralateral; Den, denervation; CHO, carbohydrate.

Supplementary Figure legends

Supplementary Figure 1: ***Correlation between decreased muscle protein synthesis and muscle weight loss due to denervation***

The data are from *n = 11* animals for the gastrocnemius (A), *n = 10 or 11* for the plantaris (B), *n = 12* for the soleus (C), n = 33 or 34 for all muscles (D) in each of the high- or low-CHO groups.

Supplementary Figure 2: ***Effects of denervation and dietary carbohydrate ratio on the total ubiquitinated protein, ubiquitin related enzyme mRNA, and 20S proteasome activity in the gastrocnemius and soleus muscle***

Relative change compared with sham-operated contralateral of high-CHO group in the total ubiquitinated protein, MUSA1 mRNA, SMART mRNA, USP19 mRNA, and 20S proteasome activity in gastrocnemius (A) and soleus (B) muscles. The data for total ubiquitin were normalized by Ponceau S staining signaling. Values are expressed as mean ± SD, and data are from *n =11 or 12* muscles per each of the high-CHO or low-CHO group. Two-way ANOVA with repeated measures was used to assess the significant effect of denervation and diet. Representative Western blots are shown on the left. Sham, sham-operated contralateral; Den, denervation; CHO, carbohydrate; NS, non-significant.

Supplementary Figure 3: ***Effects of denervation and dietary carbohydrate ratio on the intramuscular p70S6K and Akt signaling markers in the soleus muscle***

Relative change compared with sham-operated contralateral of high-CHO group

in phosphorylated p70S6K at Thr389 (A), total p70S6K (B), phosphorylated Akt at Thr308 (C), phosphorylated Akt at Ser473 (D), and total Akt (E) in the soleus muscle. Values are expressed as mean ± SD, and data are from *n = 12(A, B, C, E) or 11(D)* muscles per each of the high-CHO or low-CHO group. Two-way ANOVA with repeated measures was used to assess the significant effect of denervation and diet. Representative Western blots are shown on the left. Sham, sham-operated contralateral; Den, denervation; CHO, carbohydrate; NS, non-significant.

Supplementary Figure 4: ***Effects of denervation and dietary carbohydrate ratio on the intramuscular signaling markers for protein breakdown in the soleus muscle***

Relative change compared with sham-operated contralateral of high-CHO group in phosphorylated FoxO1 at Ser256 (A), total FoxO1 (B), phosphorylated FoxO3a at Ser253 (C), total FoxO3a (D), LC3-I (E), and LC3-II (F) in the soleus muscle. Values are expressed as mean ± SEM, and data are from *n = 11 (A, C), 12 (B, E, F), or 10(D)* muscles per each of the high-CHO or low-CHO group. Two-way ANOVA with repeated measures was used to assess the significant effect of denervation and diet. Representative Western blots are shown on the left. Sham, sham-operated contralateral; Den, denervation; CHO, carbohydrate; FoxO, Forkhead box O; LC, microtubule-associated protein light chain; NS, non-significant.

Supplementary Figure 5: ***Effects of denervation and dietary carbohydrate***

ratio on the markers for ubiquitin-proteasome pathway in the soleus muscle

Relative change compared with sham-operated contralateral of high-CHO group in Atrogin-1 protein (A), Atrogin-1 mRNA (B), and MurF-1 mRNA (C) in the soleus muscle. The data for Atrogin-1 protein were normalized by Ponceau S staining signaling. Values are expressed as mean ± SD, and data are from *n = 12 (A), n = 12 (B) or 11~12 (C)* muscles per each of the high-CHO or low-CHO group. Two-way ANOVA with repeated measures was used to assess the significant effect of denervation and diet. Representative Western blots are shown on the left. Sham, sham-operated contralateral; Den, denervation; CHO, carbohydrate; NS, non-significant.

Supplementary Figure 6: ***Effects of denervation and dietary carbohydrate ratio on intramuscular AMPK signaling markers in the soleus muscle***

Relative change compared with the sham-operated contralateral of the high-CHO group in phosphorylated ACC at Ser79 (A), total ACC (B), phosphorylated AMPK at Thr308 (C), and total AMPK (D) in the soleus muscle. Values are expressed as mean ± SD, and data are from *n = 12* muscles per each of the high-CHO or low-CHO group. Two-way ANOVA with repeated measures was used to assess the significant effect of denervation and diet. Representative Western blots are shown on the left. Sham, sham-operated contralateral; Den, denervation; CHO, carbohydrate; ACC, Acetyl-CoA carboxylase; NS, non-significant.
