# Supplementary material for: Low‐Carbohydrate Diet Exacerbates Denervation‐Induced Atrophy of Rat Skeletal Muscle Under the Condition of Identical Protein Intake
Source: J Cachexia Sarcopenia Muscle. 2025 Feb 25;16(2):e13738. doi: 10.1002/jcsm.13738 (PMC11859665; doi:10.1002/jcsm.13738)
Supplement: Supplementary file 2 — Figure S1. Correlation between decreased muscle protein synthesis and muscle weight loss due to denervation. Figure S2. Effects of denervation and dietary carbohydrate ratio on the total ubiquitinated protein, ubiquitin‐related enzyme mRNA and 20S proteasome activity in the gastrocnemius and soleus muscle. Figure S3. Effects of denervation and dietary carbohydrate ratio on the intramuscular p70S6K and Akt signalling markers in the soleus muscle. Figure S4. Effects of denervation and dietary carbohydrate ratio on the intramuscular signalling markers for protein breakdown in the soleus muscle. Figure S5. Effects of denervation and dietary carbohydrate ratio on the markers for ubiquitin–proteasome pathway in the soleus muscle. Figure S6. Effects of denervation and dietary carbohydrate ratio on intramuscular AMPK signalling markers in the soleus muscle. [file JCSM-16-e13738-s002.pdf]

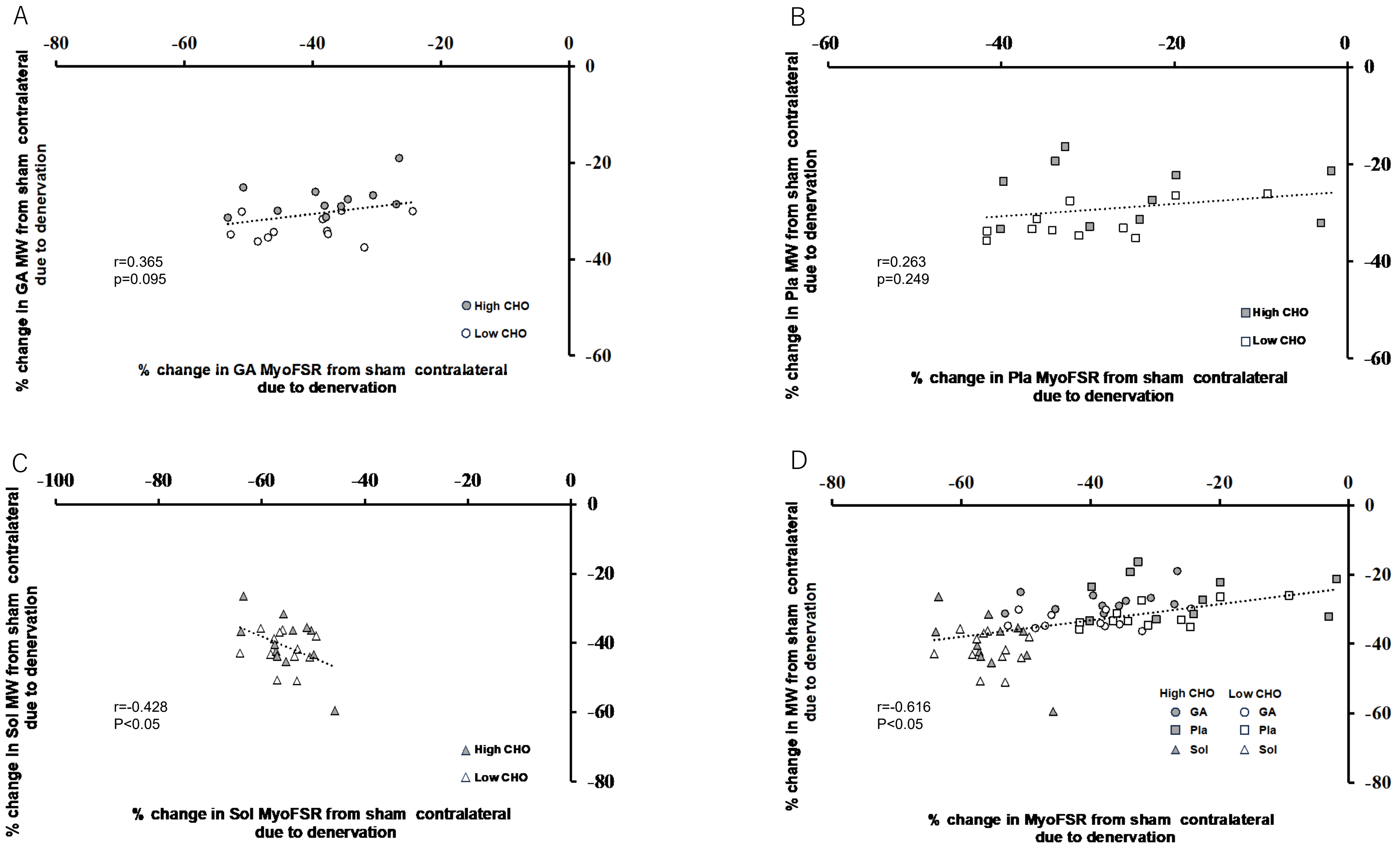

Supplementary Figure 1

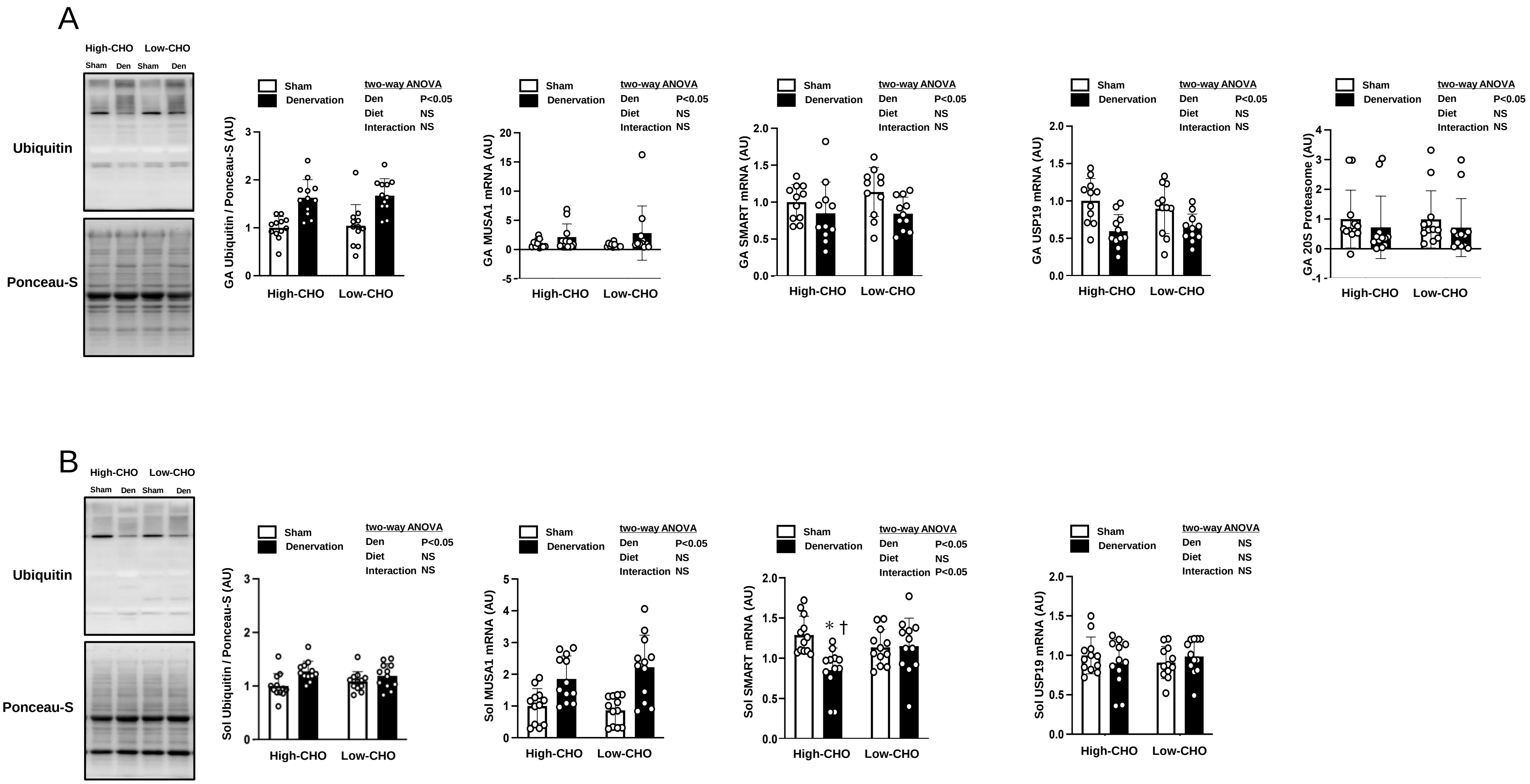

Supplementary Figure 2

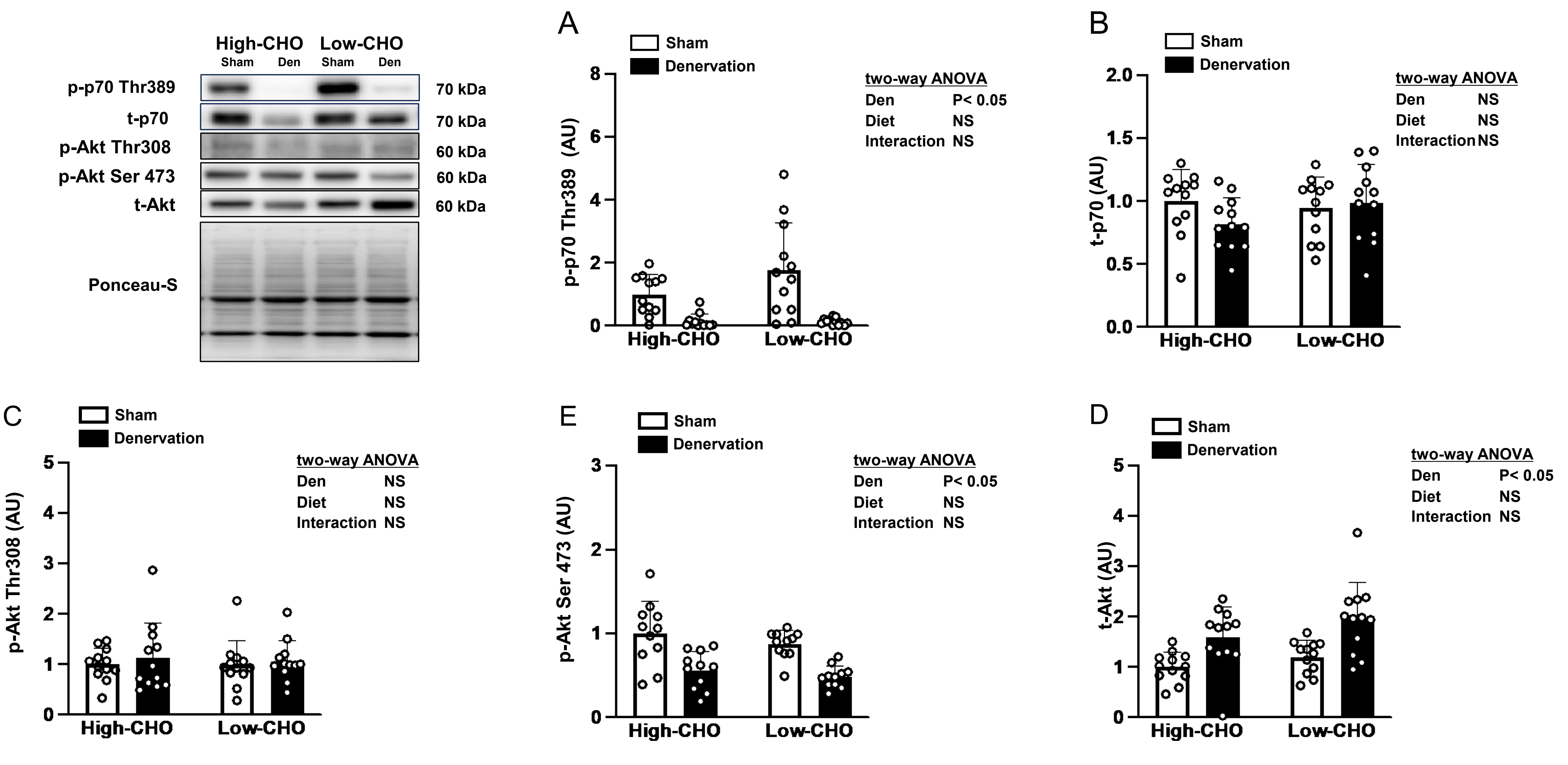

Supplementary Figure 3

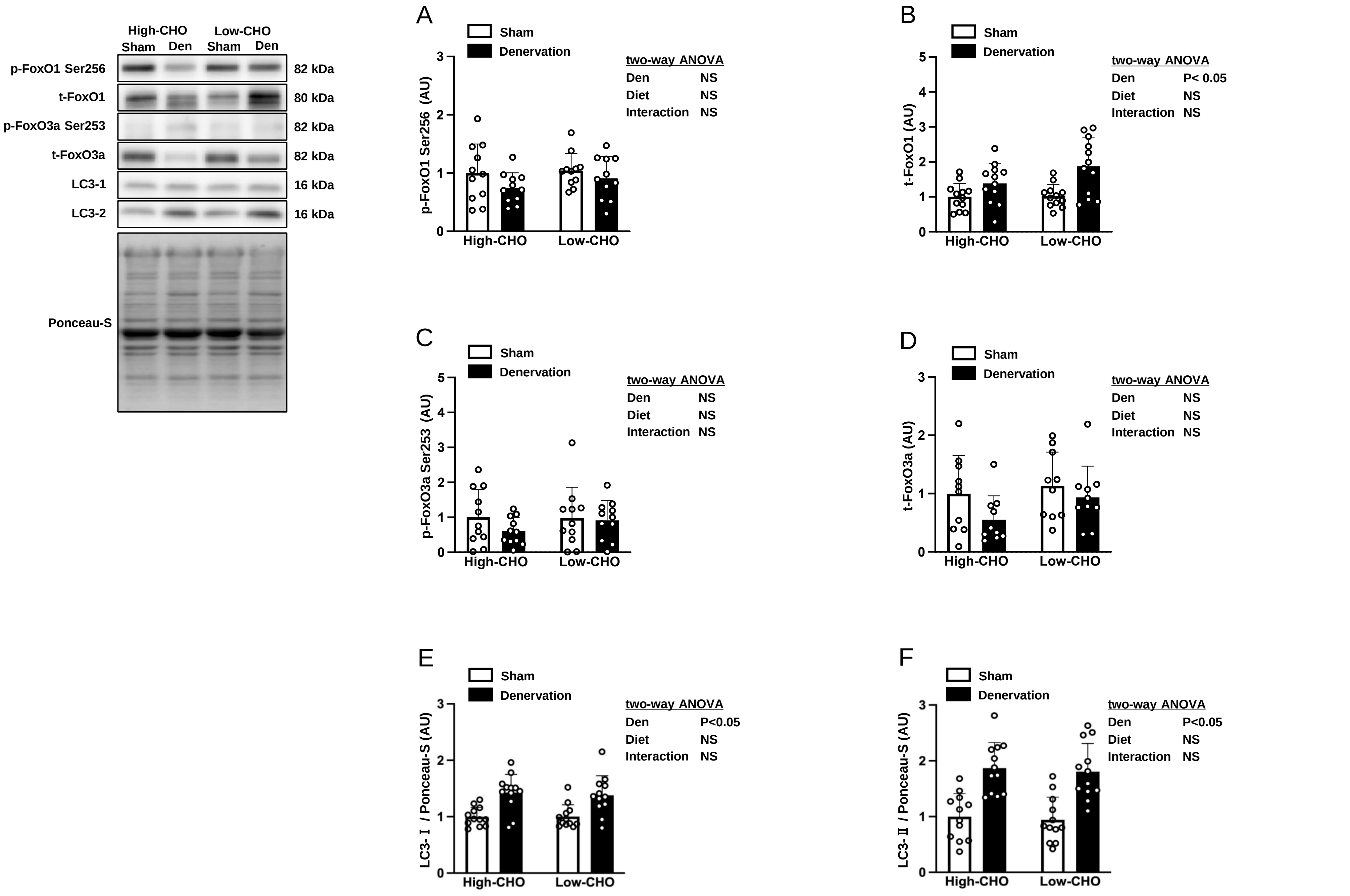

Supplementary Figure 4

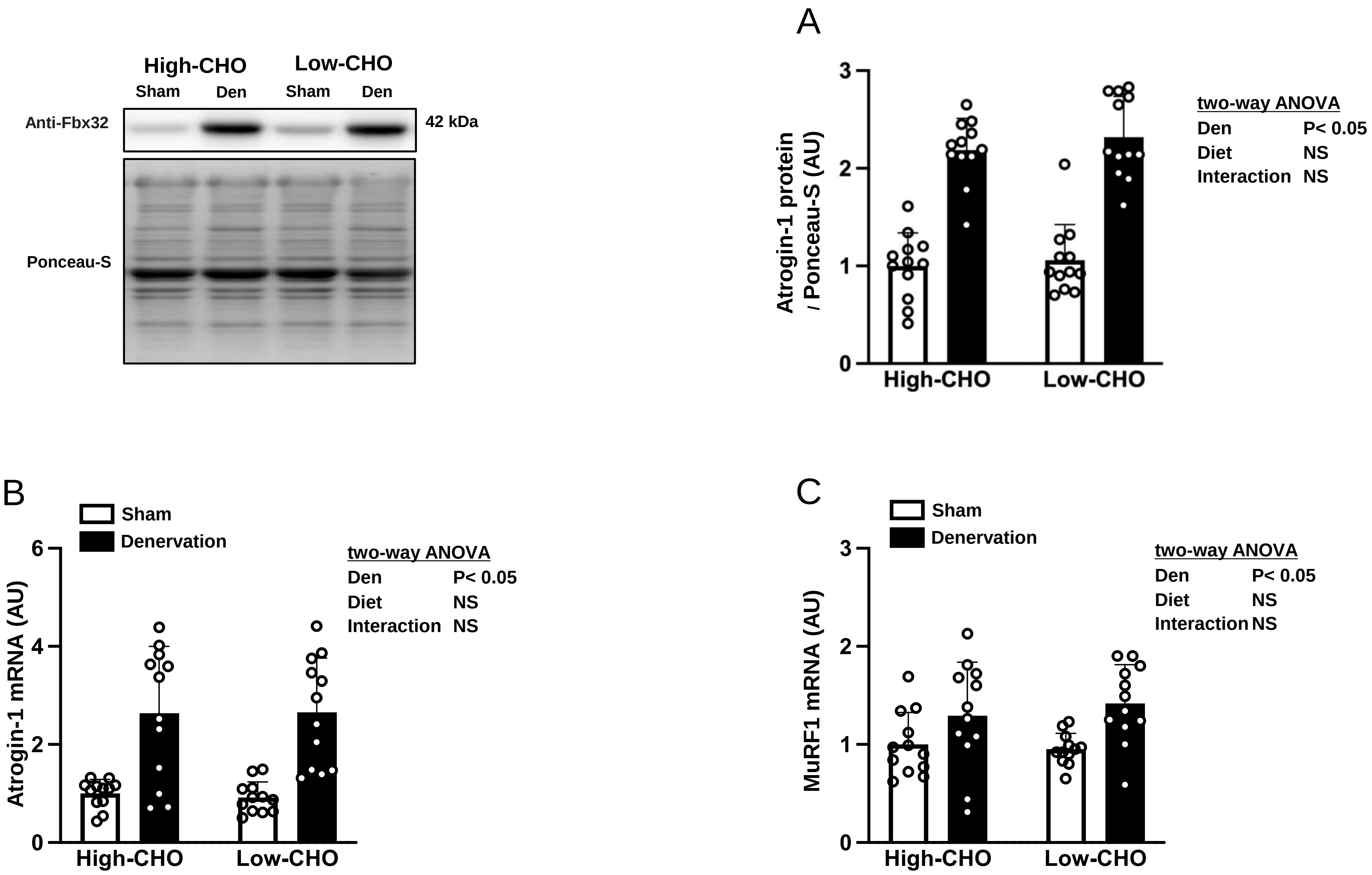

Supplementary Figure 5

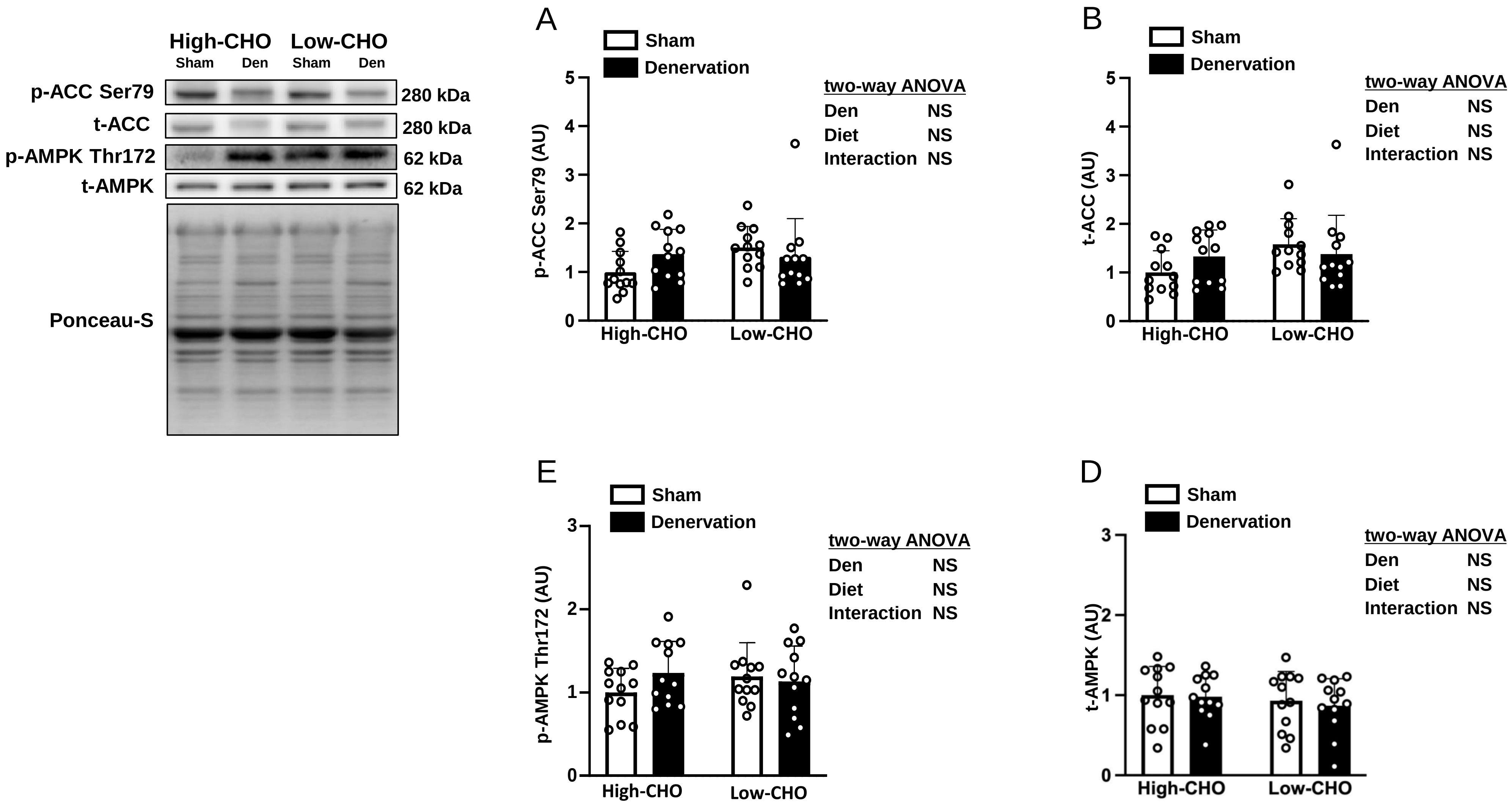

Supplementary Figure 6
